# Real-world evidence on the economic implications of CGRP-mAbs as preventive treatment of migraine

**DOI:** 10.1186/s12883-023-03302-7

**Published:** 2023-07-03

**Authors:** Nikolaj Siersbæk, Lærke Kilsdal, Christian Jervelund, Sonja Antic, Lars Bendtsen

**Affiliations:** 1 Copenhagen Economics, Langebrogade 3C, Copenhagen K, 1411 Denmark; 2 Pain and Headache Clinic, Aarhus University Hospital, Universitetsbyen 25, 1, Aarhus C, 8000 Denmark; 3 Department of Neurology, Danish Headache Center, Copenhagen University Hospital - Rigshospitalet, Valdemar Hansen Vej 5, Glostrup, 2600 Denmark

**Keywords:** CGRP-mAbs, Real-world evidence, Socioeconomic gains, Health economic savings, Indirect cost, Direct cost

## Abstract

**Background:**

Calcitonin gene-related peptide (CGRP) monoclonal antibodies (mAbs) are approved in Europe as preventive treatment of migraine in patients with at least four monthly migraine days. Migraine gives rise to direct healthcare expenditures, but most of the economic burden of migraine is socioeconomic. Evidence on the socioeconomic implications of CGRP-mAbs is, however, limited. There is an increasing interest in supplementing evidence from randomised controlled trials (RCTs) with real-world evidence (RWE) to aid clinical decision making and inform decision making for migraine management. The objective of this study was to generate RWE on the health economic and socioeconomic implications of administering CGRP-mAbs to patients with chronic migraine (CM) and episodic migraine (high-frequency episodic migraine (HFEM), and low-frequency episodic migraine (LFEM)).

**Methods:**

Real-world data (RWD) on Danish patients with CM, HFEM, and LFEM were collected via two Danish patient organisations and two informal patient networks and used in a tailored economic model. Treatment effects of CGRP-mAbs on health economic and socioeconomic outcomes were estimated using a sub-sample of patients with CM who receive CGRP-mAb treatment.

**Results:**

A total of 362 patients (CM: 199 [55.0%], HFEM: 80 [22.1%], LFEM: 83 [22.9%]) were included in the health economic model (mean age 44.1 ± 11.5, 97.5% female, 16.3% received treatment with CGRP-mAbs), and 303 patients were included in the socioeconomic model (15.2% received treatment with CGRP-mAbs). Health economic savings from initiating CGRP-mAb treatment totalled €1,179 per patient with CM per year on average (HFEM: €264, LFEM: €175). Socioeconomic gains from initiating CGRP-mAb treatment totalled an average gross domestic product (GDP) gain of €13,329 per patient with CM per year (HFEM: €10,449, LFEM: €9,947).

**Conclusion:**

Our results indicate that CGRP-mAbs have the potential to reduce both health economic expenditures and the socioeconomic burden of migraine. Health economic savings are used as a basis for health technology assessments (HTAs) of the cost-effectiveness of new treatments, which implies that important socioeconomic gains may not be given enough importance in decision making for migraine management.

**Supplementary Information:**

The online version contains supplementary material available at 10.1186/s12883-023-03302-7.

## Background

Migraine is a serious condition with debilitating symptoms [1, 2]. Migraine is second among the world’s causes of disability, and the first cause of disability in women [3]. Migraine is associated with large direct healthcare expenditures [4] and a large socioeconomic disease burden mainly driven by indirect costs from adverse implications to labour market participation, causing a lower gross domestic product (GDP) contribution [5, 6].

Calcitonin gene-related peptide (CGRP) monoclonal antibodies (mAbs) have been shown to significantly reduce the number of monthly migraine days (MMD) in patients with chronic and episodic migraine [7]. In addition, socioeconomic implications of CGRP-mAbs have been demonstrated on sick leave days [6, 8]. However, the socioeconomic implications of CGRP-mAbs in terms of GDP gain have not previously been estimated.

In several European countries, reimbursement of CGRP-mAbs is restricted to specific sub-populations within the approved indication, e.g., chronic migraine patients with at least two [9] prior treatment failures, or episodic migraine patients with at least three prior treatment failures [10]. This implies that some patients with episodic migraine and, e.g., seven MMD may not be eligible for a treatment that may help them regain up to a quarter of their life. Health technology assessments (HTAs) of new treatments focus mostly on health economic savings and quality-adjusted life years (QALYs) gained, which does not address the evidence in the literature that the majority of the burden of migraine is socioeconomic.

There is a growing interest in the use of real-world evidence (RWE) as a supplement to evidence from randomised controlled trials (RCTs) and to aid clinical decision making and inform decision makers for migraine management [11]. Real-world data (RWD) are already used in the regulation of the development, authorisation, and supervision of medicines in the European Union, and their use for demonstrating efficacy is progressing [12]. Against this background, the objective of this study was to estimate the health economic and socioeconomic implications of administering CGRP-mAbs to adult patients with chronic migraine (CM), high-frequency episodic migraine (HFEM), and low-frequency episodic migraine (LFEM).

## Methodology

A tailored economic model was developed to estimate the savings and gains from initiating treatment with CGRP-mAbs on a range of outcomes that are affected by migraine following an approach from the literature [13]. A distinction is made between health economic savings with direct implications for healthcare budgets and socioeconomic gains with indirect implications for society in terms of GDP gain.

We collected RWD on Danish adult patients with CM, HFEM, and LFEM to generate retrospective RWE on the societal implications of CGRP-mAbs. Before initiating the survey for gathering RWD, the participants provided informed consent to participate in the survey. RWD were collected through the social media networks of the Danish patient organisations, Migraine and Headache Association, and Migraine Denmark from 11 January to 7 March 2022. Followers of the two patient organisations’ social media networks do not have to be members of the patient organisations to follow them on social media. In addition, RWD were collected in the same period through the social media networks of two informal patient groups, and local physicians helped spread the word about the research study. Inclusion criteria included at least four self-reported MMD on average over the last three months or at least four self-reported MMD on average per month before initiating their current preventive treatment, at least 18 years of age, and completion of either the health economic part of the survey or both the health and socioeconomic part. CM is defined according to ICHD-3 [14]; HFEM is defined as patients reporting at least eight MMD but fewer than 15 monthly headache days (MHD), and LFEM is defined as patients with four to seven MMD [15]. Migraine attacks were defined according to the ICHD-3 classification as headache attacks lasting 4–72 hours (untreated or unsuccessfully treated) that had at least two of the following four characteristics: unilateral location, pulsating quality, moderate or severe pain intensity, and/or aggravation by or causing avoidance of routine physical activity (e.g., walking or climbing stairs), and in addition at least one of the following two characteristics: nausea and/or vomiting, or photophobia and phonophobia [14]. Patients who did not provide information on their migraine type or patients stating maximum values to all questions (e.g., 365 emergency room (ER) visits per year) were excluded.

Patients who received CGRP-mAb-treatment at the time of the data collection were asked about the implications of their migraines on their lives “today” at the time of data collection and before initiating CGRP-mAb-treatment. By comparing these two time periods, treatment effects on a broad range of clinical and non-clinical outcomes were estimated for patients with CM receiving CGRP-mAb-treatment. These were estimated both as numerical treatment effects (e.g., reduction in the number of sick days from initiation of treatment with CGRP-mAbs) and as percentage treatment effects (e.g., average percentage reduction in the number of sick days from initiation of CGRP-mAb-treatment). Baseline estimates of the same outcomes were estimated for patients with chronic migraine who did not receive treatment with CGRP-mAbs at the time of the data collection, and for patients with HFEM and LFEM who are not recommended for treatment with CGRP-mAbs in Denmark but are within the indication approved by the European Medicines Agency (EMA) [16–18]. The percentage treatment effects estimated in patients with CM who receive treatment with CGRP-mAbs were used to estimate the numerical treatment effects for CM, HFEM, and LFEM patients who do not receive treatment with CGRP-mAbs by assuming the same percentage reduction observed in patients with CM who receive CGRP-mAb-treatment [7]. This yields an estimate of the numerical reduction in health economic and socioeconomic outcomes that patients with CM, HFEM, and LFEM would receive if treatment with CGRP-mAbs was initiated.

Patients with CM may not be receiving treatment with CGRP-mAbs in Denmark because they do not fulfill the requirement to have experienced treatment failure on at least two preventive treatments (one antihypertensive and one anti-epileptic medication), have medication overuse headache, are on the waiting list to receive treatment with CGRP-mAbs, or have chosen not to initiate the treatment.

The RWD used in the health economic model include the use of acute medication, preventive medication, and healthcare resources. Healthcare resource use consists of general practitioner (GP) visits, specialist visits, outpatient visits, hospitalisations, and emergency room (ER) visits. These have all been shown to be significantly higher in patients with migraine [4, 6, 8, 19]. All measures of use of acute medication, preventive medication, and healthcare resources are based on self-reported values from the patients. Costs of medication and treatment were based on publicly available information [20, 21] and unpublished average public procurement rebates for the category of medicine used. Costs of healthcare resource use were based on collective agreements for general practitioners [22] and specialists [23] and Diagnosis Related Group (DRG) rates for hospitalisations and emergency room visit [21]; outpatient visits were based on Danish Outpatient Grouping System (Dansk Ambulant Grupperingssystem, DAGS) rates [24] projected to 2022 values using public indices [25]. Coupling the estimated treatment effects with the costs provided an estimate of the health economic savings.

The RWD used in the socioeconomic model include absenteeism (sick days), presenteeism (decreased productivity) [26], working part-time, labour market participation, educational attainment, and career choice. These have all been shown to be important implications for people with migraine [6, 8, 9, 27–30]. Absenteeism was measured using Item 2 of the Work Productivity and Activity Impairment (WPAI) instrument [31], and presenteeism was measured by the Stanford Presenteeism Scale SPS-6 [26]. Working part-time was measured as the share wanting to work more hours, and if so, how many hours. Labour market participation was measured as the share that would not have left the labour market had they received treatment with CGRP-mAbs. Educational attainment and career choice were measured as the share of patients who would have obtained a different education or pursued a different career had they received treatment with CGRP-mAbs. The average potential earnings from avoiding working part-time due to migraine were estimated from the patients’ self-reported earnings. For labour market participation, average income levels by educational level were used. For higher educational attainment, the expected increase in earnings by comparing patients’ self-reported earnings with the average earnings from higher educational attainment was used. Lastly, for career choice, patients’ self-reported expected increase in earnings had they been able to pursue the career was used in combination with patients’ self-reported earnings.

The savings and gains are reported as the averages per patient initiating treatment according to the Danish guidelines [9]. Health economic savings and socioeconomic gains were estimated as averages across all patients initiating treatment and adjusted to reflect that only a subset of patients initiating treatment respond. A recent study performed in 300 Danish CM patients demonstrated that 71.4% of patients initiating treatment with CGRP-mAbs responded with at least a 30% reduction in MMD after 12 weeks [32], allowing treatment to be continued according to Danish guidelines [9]. The same study showed that 56.4% of patients experienced a 50% reduction in MMD, which is higher than what is previously found in clinical studies [33], indicating that experiences from RWE show larger effects on patients than RCTs. The estimated health economic savings and socioeconomic gains reported here thus consist of savings and gains from the 71.4% of patients who respond, averaged across all patients that initiate treatment.

All monetary values are reported in Euros, using a conversion rate from Danish Kroner (DKK) (original currency) to Euros of €0.1344 per DKK. The statistical analysis was carried out in StataIC 15.1. Statistical significance was estimated using two-sided paired Student’s t-tests on the null hypothesis of equal means for sub-samples with at least 30 respondents. For sub-samples with fewer than 30 patients, Wilcoxon signed-rank test on the null hypothesis of no difference in population mean ranks was used.

## Results

A total of 665 individuals initiated the survey. Of these, 303 patients were excluded due to the following: only looking at the front page (83), self-identifying as having fewer than four monthly migraine days (142), failing to complete the survey (62), and being omitted in data cleaning (16). The dataset for the health economic analysis totaled 362 patients, whereof 59 (16.3%) received treatment with CGRP-mAbs at the time of the data collection. Additionally, 59 patients failed to complete the questions for the socioeconomic model, totaling 303 patients in the socioeconomic analysis. Of these, 46 (15.2%) received treatment with CGRP-mAbs at the time of the data collection. Descriptive characteristics for the full sample size (N = 362) included in this study are shown in Table 1.

**Table 1:** Descriptive statistics

**PANEL A**				
**Variable**	**Category**	**Mean**	**SD**	**n**
Age		44.05	11.46	359
Earnings (EUR)^a^		46,739	27,185	335
MMD	CM^b^	12.95	7.09	199
	HFEM^c^	10.45	3.49	80
	LFEM^d^	5.20	1.08	83
MHD	CM	18.38	7.76	197
	HFEM	12.76	5.47	80
	LFEM	7.96	4.35	83
**PANEL B**				
**Variable**	**Category**		**Number (%)**	**N** ^**e**^
Gender	Female		350 (97.5%)	359
	Male		9 (2.5%)	359
Education	Elementary school		13 (3.6%)	362
	Qualifying education		2 (0.6%)	362
	Gymnasium		23 (6.4%)	362
	Vocational training		55 (15.2%)	362
	Short higher education		23 (6.4%)	362
	Bachelor’s degree		60 (16.6%)	362
	Medium higher education		91 (25.1%)	362
	Long higher education		92 (25.4%)	362
	Other		3 (0.8%)	362
Employment	Full-time		126 (34.9%)	361
	Self-employed		23 (6.4%)	361
	Part-time		96 (26.6%)	361
	Student		29 (8.0%)	361
	Unemployed		11 (3.0%)	361
	Not active in the labour market		52 (14.4%)	361
	Other		24 (6.6%)	361
Civil status	Married		188 (52.1%)	361
	In a relationship		97 (26.9%)	361
	Single		73 (20.2%)	361
	Does not want to answer/other		3 (0.8%)	361
Migraine type	CM		199 (55.0%)	362
	HFEM		80 (22.1%)	362
	LFEM		83 (22.9%)	362
Share receiving treatment with CGRP-mAbs			16.3%	362
Share receiving other preventive treatment, disregarding the CGRP-mAb-treatment group			33.3%	303

*SD* standard deviation; *MHD* monthly headache days; *MMD* monthly migraine days; *CGRP* calcitonin gene-related peptide; *mAbs* monoclonal antibodies.

^a^ Patients with missing information on earnings are assigned the average earnings from Statistics Denmark based on their gender and educational attainment. This implies that all 362 respondents have average earnings of €46,919 (SD = 26,879).

^b^ CM is defined according to ICHD-3 [14].

^c^ HFEM is defined as patients reporting at least eight MMD but fewer than 15 MHD [15].

^d^ LFEM is defined as patients with four to seven MMD [15].

^e^ The number of respondents in the total sample for which the shares are calculated differs marginally as some respondents did not answer all questions, e.g., on gender and employment.

Patients who received treatment with CGRP-mAbs at the time of the data collection reported a 34.92% reduction in MMD (from 16.36 to 10.64, difference = 5.71, p-value < 0.001, confidence interval (CI) = 4.19–7.23) compared to before they initiated treatment.

### Treatment effect

The estimated treatment effects in the health economic model show significant reductions in a wide range of outcomes, as shown in Table 2. The average use of simple analgesics before initiating treatment with CGRP-mAbs was 10.10 tablets per patient per month, and 4.58 tablets per patient per month on average after initiation of CGRP-mAb-treatment (difference = 5.53, p = 0.054, CI = -0.10-11.15). The average use of acute medication, e.g., triptan tablets, was significantly reduced (difference = 3.49 fewer tablets per patient per month on average, p < 0.001, CI = 2.21–4.78) with an estimated treatment effect of 45.7% reduction after initiation of treatment with CGRP-mAbs. Note that all estimates are averages across all patients, irrespective of whether they use the specific medication or not, to obtain an estimate of the treatment effects on an average patient. The average use of preventive medication, e.g., botulinum toxin, was significantly reduced (difference = 0.22 fewer treatment cycles per patient on average, p = 0.041, CI = 0.01–0.43) after initiation of treatment with CGRP-mAbs, which follows treatment guidelines in Denmark that preclude the simultaneous use of CGRP-mAbs and botulinum toxin [9]. Significant reductions in healthcare use were also found. The average number of migraine-related GP visits decreased from 2.57 visits per year on average to 1.76 visits per year on average (difference = 0.81, p = 0.020, CI = 0.13–1.49). Similar effects were found for other healthcare use.

**Table 2:** Treatment effects in the health economic model

Variable	Average value pre-treatment	Average value post-treatment	Difference (CI) [SD]	Difference (%)	p-value	n	Number receiving treatment, pre-treatment | post-treatment
Simple analgesics (tablets per month)^a^	10.10	4.58	5.53 (-0.10–11.15) [21.60]	54.7%	0.054	59	30 | 26
Antiemetic tablets (tablets per month)	2.69	0.90	1.80 (0.82–2.77) [3.74]	66.7%	0.000	59	26 | 14
Antiemetic injections (injections per month)	0	0.02	+ 0.02 (-0.05–0.02) [0.130]	-	0.322	59	0 | 1
Treo (tablets per month)^b^	7.25	3.75	3.51 (0.90–6.11) [10.00]	48.4%	0.001	59	25 | 25
Triptan tablets (tablets per month)	7.64	4.15	3.49 (2.21–4.78) [4.94]	45.7%	0.000	59	40 | 38
Triptan melt tablets (melt tablets per month)	1.81	0.76	1.05 (-0.03–2.13) [4.15]	57.9%	0.057	59	10 | 11
Triptan nose spray (uses per month)	0.31	0.31	0 (-0.34–0.34) [1.30]	0%	1.000	59	2 | 3
Triptan injections (injections per month)	0.02	0.02	0 (-0.05–0.05) [0.19]	0%	1.000	59	1 | 1
Beta-blockers (tablets per month)	10.42	1.61	8.81 (3.30–14.33) [21.16]	85.6%	0.002	59	14 | 6
Antihypertensive (other than beta-blockers, tablets per month)	15.58	7.41	8.17 (3.08–13.26) [19.53]	52.4%	0.002	59	24 | 12
Botulinum toxin (treatment cycles per month)	0.32	0.10	0.22 (0.01–0.43) [0.81]	68.8%	0.041	59	17 | 3
Anti-depressants (tablets per month)	9.73	5.66	4.07 (-0.81–9.95) [22.56]	41.8%	0.171	59	14 | 8
Anti-epileptics (tablets per month)	13.44	2.63	10.81 (4.88–16.75) [22.77]	80.5%	0.000	59	17 | 3
GP visits (visits per year)	2.57	1.76	0.81 (0.13–1.49) [2.57]	32.7%	0.020	58	37 | 26
Specialist visits (visits per year)	1.97	1.12	0.85 (0.17–1.53) [2.61]	43.1%	0.015	59	31 | 20
Outpatient visits (visits per year)	4.07	3.51	0.56 (-1.41–2.53) [7.56]	13.8%	0.572	59	43 | 54
ER visits (visits per year)	0.59	0.25	0.34 (0.05–0.63) [1.09]	57.1%	0.020	59	11 | 6
Cost of hospitalisation^c^ (cost of hospitalisation per year)	5,780	801	4,979 (1,141–8,816) [14,725]	13.9%	0.012	59	14 | 3

*CI* confidence interval; *SD* standard deviation; *GP* general practitioner; *ER* emergency room. Questions on medication is the use of medication in a month. Questions on healthcare resource use is annual visits. 95% confidence intervals are reported in parentheses, and the standard deviation is reported in brackets. Statistical significance was estimated using a two-sided paired Student’s t-test on the null hypothesis of equal means pre and post treatment.

^a^ Simple analgesics include non-steroidal anti-inflammatory drugs (NSAIDs) and paracetamol.

^b^ Treo is a combination analgesic containing 500 mg acetylsalicylic acid and 50 mg caffeine.

^c^ Hospitalisation is reported as a cost, as the cost depends on the length of stay *and* the number of hospitalisations [21]. Respondents were asked about the number and length of stays, which allows for the calculation of the cost of hospitalisations.

The estimated treatment effects in the socioeconomic model show large reductions in adverse socioeconomic outcomes, as shown in Table 3. For example, an 18.8% reduction is found in presenteeism using the normalised SPS-6 score ranging between 0 and 1 (difference in normalised SPS-6 = 0.14, p = 0.004). Also, a 49.83% change in labour market participation is found, indicating that amongst patients receiving treatment with CGRP-mAbs who are not active in the labour market due to early retirement or receiving disability benefits who would still like to be active in the labour market, treatment with CGRP-mAbs increased the likelihood of labour market participation by 49.83% (p = 0.026).

**Table 3:** Treatment effects in the socioeconomic model

Variable	Average value pre-treatment	Average value post-treatment	Difference	Difference (%)	p-value	n
Sick days (hours lost)^a^	5.00	3.59	1.41	28.2%	0.120	29
Presenteeism^b^	0.73	0.59	0.14	18.8%	0.004	29
Working part-time (hours worked)^c^	22.00	25.88	+ 3.88	17.6%	0.314	17
Labour market participation^d^	49.83%		-	-	0.026	6
Educational attainment^d^	35.31%		-	-	0.002	13
Career choice^d^	39.05%		-	-	0.000	19

Statistical significance was estimated using Wilcoxon signed-rank test on the null hypothesis of no difference in population mean ranks.

^a^ Sick days are measured b they Working Productivity and Activity Impairment (WPAI) Item 2 [31]. In Item 2, respondents are asked about hours lost at work in the previous seven days due to migraine.

^b^ Presenteeism is measured by the Standard Presenteeism Scale (SPS-6) [26]. In this study, the scoring is inverted and normalised to a scale between 0 and 1.

^c^ Measured by the weekly hours worked.

^d^ For labour market participation, educational attainment, and career choice, before and after values and hence differences cannot be calculated.

The estimated differences in Tables 2 and 3 are used as treatment effects from initiating CGRP-mAb-treatment. Not all treatment effects are statistically significant, likely due to the small sample size; see Discussion.

### Health economic savings

**Fig. 1:**
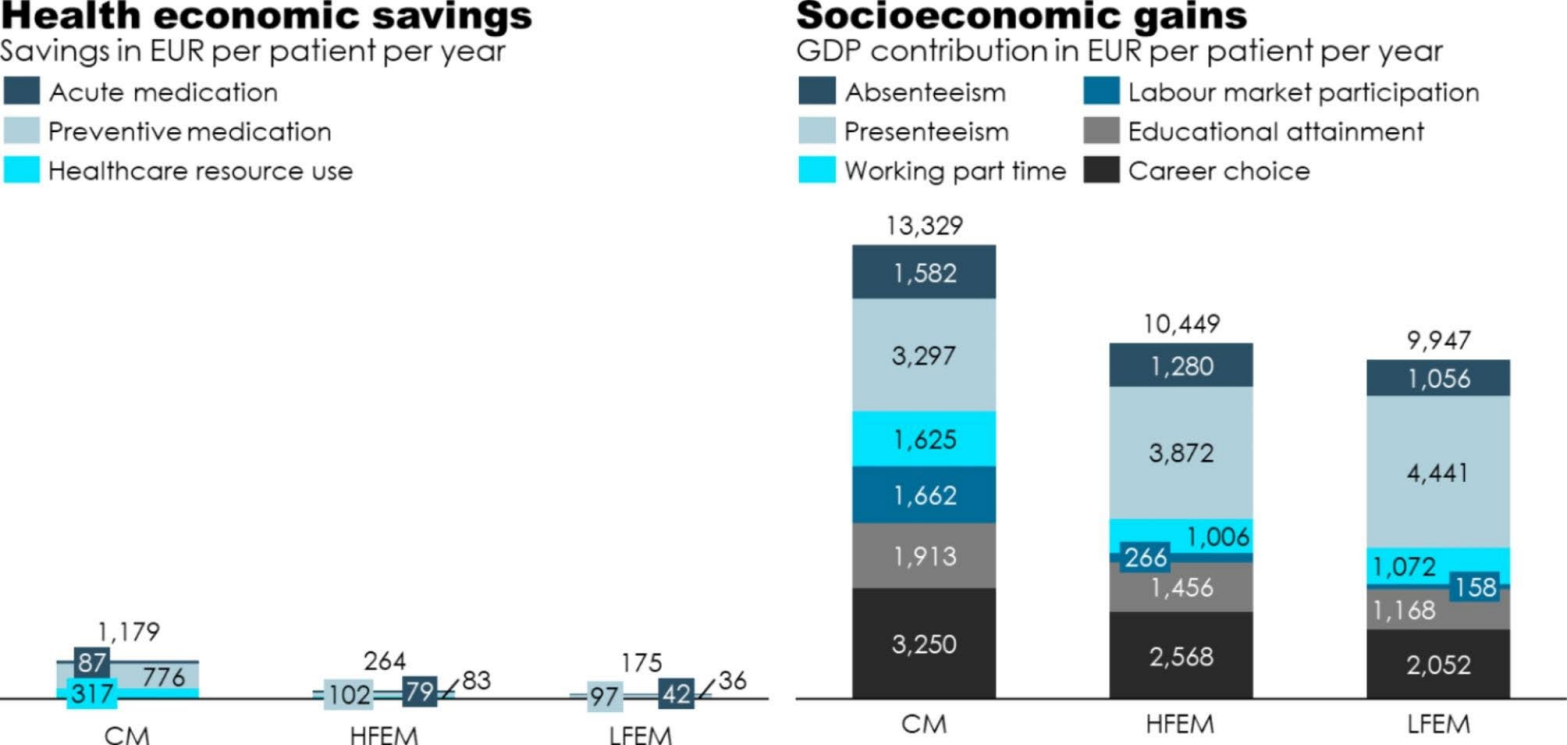
Health economic savings and socioeconomic gains associated with CGRP-mAbs *CM* Chronic migraine; *HFEM* High-frequency episodic migraine; *LFEM* Low-frequency episodic migraine; *GDP* Gross domestic product

Our estimates of the total health economic savings from initiating treatment with CGRP-mAbs in patients with CM, HFEM, and LFEM totalled €1,179, €264, and €175 per patient per year on average, respectively, as shown in Table 4 and illustrated in Fig. 1. Across all three migraine types, preventive medication comprises the largest component of health economic savings (€97 – €776). The large saving for patients with CM was mainly driven by savings for treatment with botulinum toxin despite the low number of average injections per month, due to the comparatively high cost of botulinum toxin. The second highest component is healthcare resource use for patients with CM and HFEM. The second highest saving for patients with LFEM is acute medication.

**Table 4:** Health economic savings, EUR per patient initiating treatment per year

	Chronic migraine		High-frequency episodic migraine		Low-frequency episodic migraine	
	Baseline	Savings	Baseline	Savings	Baseline	Savings
Attack medication	350	87	245	79	130	42
Preventive medication	1,808	776	178	102	164	97
Healthcare resource use	1,007	317	287	83	150	36
**Total**	**3,164**	**1,179**	**709**	**264**	**444**	**175**

Totals may not correspond with the sum of the separate figures due to rounding

#### Socioeconomic gains

Our estimates of the total socioeconomic gains from initiating treatment with CGRP-mAbs in patients with CM, HFEM, and LFEM totalled an average GDP gain of €13,329, €10,449, and €9,947 per patient per year on average, respectively, as shown in Table 5 and illustrated in Fig. 1. Across CM, HFEM, and LFEM, presenteeism comprises the largest socioeconomic gain. The gain from less presenteeism is largest for patients with LFEM as the share active of the labour market is largest amongst this group of patients.

**Table 5:** Socioeconomic gains, EUR GDP contribution per patient initiating treatment per year

	Chronic migraine		High-frequency episodic migraine		Low-frequency episodic migraine	
	Baseline	Gain	Baseline	Gain	Baseline	Gain
Absenteeism	6,325	1,582	5,118	1,280	4,223	1,056
Presenteeism	24,624	3,297	28,922	3,872	33,171	4,441
Working part-time	12,905	1,625	7,995	1,006	8,515	1,072
Labour market participation	5,723	1,662	4,324	266	1,470	158
Educational attainment	24,633	1,913	34,651	1,456	39,083	1,168
Career choice	27,579	3,250	37,224	2,568	53,765	2,052
**Total**	**101,791**	**13,329**	**118,233**	**10,449**	**140,227**	**9,947**

Baseline values for these parameters are the average earnings for patients replying “yes” to the question, scaled to an average for all patients. I.e., if patients working part-time earn an average of €50,000 and 50% are working part-time, the average effect across all patients is €50,000 * 50% = €25,000. *GDP* Gross domestic product.

Totals may not correspond with the sum of the separate figures due to rounding

## Discussion

We found health economic savings from initiation of treatment with CGRP-mAbs of €1,179, €264, and €175 for patients with CM, HFEM, and LFEM, respectively. Socioeconomic gains from initiating treatment with CGRP-mAbs amounted to €13,329 €10,449, and €9,947 for patients with CM, HFEM, and LFEM, respectively. For comparison, an estimate of the confidential annual net price of CGRP-mAbs in Denmark is approximately €3,562 per patient per year, and €4,131 per patient per year taking the extra outpatient visits associated with the treatment into account [9]. This estimate is based on the average list price of CGRP-mAb-treatments available in Denmark and an average discount for similar products negotiated by the Danish procurer of hospital medicine in Denmark.

Our results indicate that socioeconomic gains are important drivers for the societal gains associated with CGRP-mAb-treatment. This is in line with Sussman et al. (2018) and Autio et al. (2021), both of whose studies find substantial socioeconomic gains associated with treatment with CGRP-mAbs [6, 8]. Sussman et al. (2018) use Monte Carlo patient simulation and Markov cohort model and find that the treatment is more cost-effective from a societal perspective than from a payer perspective, indicating that the socioeconomic gains are significant and worth taking into account when evaluating the treatment. Likewise, Autio et al. (2021) find that treatment with CGRP-mAbs decreases not only healthcare visits but also sick leave days [8]. Our results also indicate that socioeconomic gains make up 92–98% of the total gains related to treatment with CGRP-mAbs, highlighting that societal perspectives are important to consider when assessing diseases that affect the working-age population. These results are in line with Linde et al. (2012), who find that 93% of the burden of migraine stems from socioeconomic implications [5].

Socioeconomic implications of new treatments are most often not explicitly considered in health technology assessments (HTAs) in Europe, and productivity effects are excluded [34]. Our results thus indicate that the majority of the economic benefits of CGRP-mAb-treatment are not included in the evaluation of current treatments.

The present study design has some limitations. The sample was a non-random sample of patients who self-selected into participation in the research project, which may give rise to self-selection bias [35]. Even though respondents did not have to be members of patient organisations to participate, they needed for follow the social media networks of one of the two patient organisations or the two informal patient networks. The RWD are based solely on patient-reported data, which may have lower reliability and validity than other objective data sources [36]. The retrospective setup could be subject to biases including recall bias and overestimation of the severity of adverse outcomes [37]. The RWD were collected through a web-based survey with potential under-coverage bias from specific groups of patients being under-represented because they have less access to the Internet [35]. In Denmark, 97% of families have Internet access at home [38], but this share is lower for elderly people, who thus may be under-represented in the RWD. The treatment effects are at most based on 59 patients with CM who received CGRP-mAb-treatment at the time of the RWD collection. As mentioned, only patients with CM with at least two prior treatment failures are recommended for reimbursement of CGRP-mAbs in Denmark [9]. Different sub-analyses of, e.g., implications for labour market participation, are based on sub-samples of these patients, which gives rise to small-sample issues including a lack of statistical significance. There are currently approximately 1,500 patients with chronic migraine who receive treatment with CGRP-mAbs in Denmark. A power analysis using Lehr’s rule of thumb [39] shows that the sample size needed to obtain statistical significance of the treatment effect of, e.g., sick days at the 5% level is 748 patients ($$16*{9.64}^{2}/{1.41}^{2})$$, i.e., 49.9% of the total patient population, which is unlikely to be obtained using the data collection process we used in the present study.

A total of 97.5% of patients in the RWD are female, which is higher than prevalence estimates would suggest [40]. This may be driven by the collection of RWD through social media networks since women are more engaged in using the Internet for health-related information searching [41]. The large share of women in our sample can have implications for its representativeness of the population of patients with CM, HFEM, and LFEM in Denmark and thus the estimated economic implications. Importantly, our estimates could be biased if (a) healthcare resource utilisation for women with specific types of migraine (e.g., CM) is markedly different from utilisations by men with the same type of migraine, or (b) labour market outcomes for women are markedly different from outcomes for men. We believe our estimates are a fair representation of the effect of CGRP-mAbs in the Danish population of patients with migraine for two reasons. First, while sex-related differences in the epidemiology, clinical features, and pathophysiology of migraine have been shown [42], no data on sex-related differences in costs among patients with migraine in Denmark is available to our knowledge, and there is no clear bias in the related literature. A Danish population study has found lower contact rate to GPs, but higher hospitalisation for men than women, which does not provide a clear direction of the bias in a Danish context [43]. Similarly, a large registry-based study in diabetes found on average higher healthcare costs among men than women [44]. An Italian study found that men consult a doctor less often than women for treatment, whereas women are more likely to talk with their primary provider or contact a headache centre [45, 46]. Compared with men, women have been found to use more prescription medications and are more likely to use triptans or drug combinations [42, 45, 47, 48]. All of these mechanisms – if applicable in a Danish context – may bias our estimates upward due to the large share of women. Second, the overrepresentation of women may have a dampening effect on our estimated socioeconomic gains due to the remaining gap in average earnings between men and women in Denmark[49]. As a result, our socioeconomic results can be interpreted as a conservative estimate for the population of patients with CM, HFEM, and LFEM.

The percentage treatment effects estimated in patients with CM who receive treatment with CGRP-mAbs are used to estimate the numerical treatment effects in patients with CM, HFEM, or LFEM who do not receive treatment with CGRP-mAbs by assuming the same percentage reduction that is observed in patients with CM who receive CGRP-mAb-treatment. There is no formal test of this assumption, but to show the comparability of patients with CM who receive treatment with CGRP-mAbs to patients with CM who do not, descriptive statistics split between patients receiving treatment with CGRP-mAbs and patients with CM not receiving treatment with CGRP-mAbs are summarised in Supplementary Table S1 The two groups are generally comparable in terms of their descriptive characteristics despite some differences in educational attainment where a higher share of patients receiving treatment with CGRP-mAbs have a bachelor’s degree, and lower shares have vocational training or a long higher education. Despite this difference, the comparison indicates that there is no selection bias in terms of observable characteristics of the patients and that the percentage of treatment effects estimated in patients with CM who receive treatment with CGRP-mAbs can be generalised to other patients with CM. The effect of treatment becomes apparent when examining Table S1 as patients not receiving treatment with CGRP-mAbs experienced more MMD and MHD than patients receiving treatment. Compared to patients with HFEM and LFEM, patients with CM had on average shorter education and were more likely to work part-time, cf. Table S1. This difference and other differences on baseline values was controlled for by estimating the average gain per person. This implies that a gain of, e.g., €5,000 for one working individual is reduced to an average gain of €1,250 per person if only 25% are working. The differences in education and employment across migraine types are controlled for by using the specific shares of working part-time, active in the labour market, and employed from the survey. In addition to the indications above, clinical trials with primary endpoints on MMD reduction and share of patients responding indicate that it may be reasonable to apply percentage treatment effects estimated in patients with CM to patients with HFEM and LFEM [7]. Due to the above-mentioned limitations, the exact economic savings and gains from treatment with CGRP-mAbs may differ from our results. However, the suggested economic savings and gains presented here are our best estimates at present. Even though the true savings and gains may deviate from our results, we believe these results are strong indications that the economic savings and gains from treating migraine patients with CGRP-mAbs are substantial.

The reimbursement practice in Denmark did not enable us to analyse the potential of CGRP-mAbs to prevent chronification of migraine from episodic migraine (LFEM or HFEM) to CM, since only patients with CM are eligible for treatment. An addition, the observed implications of CM, HFEM, and LFEM on, e.g., educational attainment and being active in the labour market (cf. Table S1) may not be limited to patients with four or more MMD but could be relevant for patients with fewer MMD who are not within the indication for CGRP-mAbs. We see these as opportunities for future research.

## Conclusion

Our results indicate that CGRP-mAbs have the potential to reduce both the direct health economic expenditures and the socioeconomic burden of migraine, thus having significant labour market implications. Further research is needed to confirm the health and socioeconomic implications of CGRP-mAbs in patients with CM, HFEM, and LFEM using, e.g., longitudinal research designs. Health technology assessments currently use health economic savings as a basis for evaluating the cost-effectiveness of new treatments, which implies that important societal gains in the form of socioeconomic savings may not be given enough importance in decision making.

## Electronic supplementary material

Below is the link to the electronic supplementary material.

Supplementary Material 1

## Data Availability

The dataset generated and analysed during the current study are not publicly available because the dataset contains personal information collected for the purpose of this study. Due to General Data Protection Regulation (GDPR), the authors are prevented from sharing the raw data with other parties. In case of additional requests, please contact the corresponding author.

## Declarations

### Authours’ information

NS, LK, and CJ are employees of Copenhagen Economics, which is one of the leading economics firms in Europe. Founded in 2000, we currently have more than 100 employees operating from our offices in Copenhagen, Stockholm, Helsinki, and Brussels. The Global Competition Review (GCR) lists Copenhagen Economics among the top 20 economic consultancies in the world, and has done so since 2006.

## Abbreviations

CI: Confidence Interval
CM: Chronic migraine
CGRP: Calcitonin gene-related peptide
DAGS: Danish outpatient grouping system (Dansk ambulant grupperingssystem)
DKK: Danish Kroner
DRG: Diagnosis related groups
EMA: European Medicines Agency
ER: Emergency room
EUR: Euro
GDP: Gross domestic product
GP: General practitioner
HFEM: High-frequency episodic migraine
HTAs: Health technology assessments
LFEM: Low-frequency episodic migraine
mAbs: Monoclonal antibodies
MHD: Monthly headache days
MMD: Monthly migraine days
QALYs: Quality-adjusted life years
RCT: Randomised controlled trials
RWD: Real-world data
RWE: Real-world evidence
WPAI: Work Productivity and Activity Impairment
