# Supplementary material for: Real-world evidence on the economic implications of CGRP-mAbs as preventive treatment of migraine
Source: BMC Neurol. 2023 Jul 3;23:254. doi: 10.1186/s12883-023-03302-7 (PMC10316628; doi:10.1186/s12883-023-03302-7)
Supplement: Supplementary file 1 — Supplementary Material 1 [file 12883_2023_3302_MOESM1_ESM.docx]

**Real-world evidence on the economic implications of CGRP-mAbs as preventive treatment of migraine**

# Supplementary material

**Table S.1 Descriptive statistics for patients with CM receiving treatment with CGRP-mAbs, other patients with CM, and patients with HFEM and LFEM**

| PANEL A | | | | | | | | |
| --- | --- | --- | --- | --- | --- | --- | --- | --- |
|  | **CM receiving CGRP-mAbs** | | **Other CM** | | **HFEM** | | **LFEM** | |
| Variable | **Mean (SD)** | **n** | **Mean (SD)** | **n** | **Mean (SD)** | **n** | **Mean (SD)** | **n** |
| Age | 46.03 (11.04) | 58 | 43.47 (12.66) | 139 | 44.39 (9.93) | 80 | 43.30 (11.00) | 82 |
| Earnings (EUR) | 45,094 (26,815) | 59 | 42,273 (24,840) | 140 | 47,498 (26,088) | 80 | 55,496 (29,263) | 83 |
| MMD | 10.64 (7.14) | 59 | 13.92 (6.87) | 140 | 10.45 (3.49) | 80 | 5.20 (1.08) | 83 |
| MHD | 14.68 (7.97) | 57 | 19.89 (7.18) | 140 | 12.76 (5.47) | 80 | 7.96 (4.35) | 83 |

| PANEL B | | | | | |
| --- | --- | --- | --- | --- | --- |
|  |  | **CM receiving CGRP-mAbs** | **Other CM** | **HFEM** | **LFEM** |
| Variable |  | **n (%)^a^** | **n (%)^a^** | **n (%)^a^** | **n (%)^a^** |
| Gender | Female | 57 (98.3%) | 136 (97.8%) | 79 (98.8%) | 78 (95.1%) |
|  | Male | 1 (1.7%) | 3 (2.2%) | 1 (1.3%) | 4 (4.9%) |
| Education | Elementary school | 1 (1.7%) | 8 (5.7%) | 4 (5.0%) | 0 (0.0%) |
|  | Qualifying education | 0 (0.0%) | 1 (0.7%) | 1 (1.3%) | 0 (0.0%) |
|  | Gymnasium | 5 (8.5%) | 11 (7.9%) | 4 (5.0%) | 3 (3.6%) |
|  | Vocational training | 8 (13.6%) | 26 (18.6%) | 11 (13.8%) | 10 (12.0%) |
|  | Short higher education | 8 (13.6%) | 3 (2.1%) | 9 (11.3%) | 3 (3.6%) |
|  | Bachelor’s degree | 13 (22.0%) | 23 (16.4%) | 13 (16.3%) | 11 (13.3%) |
|  | Medium-term higher education | 17 (28.8%) | 39 (27.9%) | 19 (23.8%) | 16 (19.3%) |
|  | Long higher education | 7 (11.9%) | 28 (20.0%) | 18 (22.5%) | 39 (47.0%) |
|  | Other | 0 (0.0%) | 1 (0.7%) | 1 (1.3%) | 1 (1.2%) |
| Employment | Full-time | 12 (20.3%) | 34 (24.3%) | 36 (45.6%) | 44 (53.0%) |
|  | Self-employed | 3 (5.1%) | 9 (6.4%) | 6 (7.6%) | 5 (6.0%) |
|  | Part-time | 21 (35.6%) | 34 (24.3%) | 16 (20.3%) | 25 (30.1%) |
|  | Student | 4 (6.8%) | 14 (10.0%) | 7 (8.9%) | 4 (4.8%) |
|  | Unemployed | 2 (3.4%) | 6 (4.3%) | 3 (3.8%) | 0 (0.0%) |
|  | Not active in the labour market | 10 (16.9%) | 32 (22.9%) | 7 (8.9%) | 3 (3.6%) |
|  | Other | 7 (11.9%) | 11 (7.9%) | 4 (5.1%) | 2 (2.4%) |
| Civil status | Married | 34 (57.6%) | 66 (47.1%) | 51 (63.8%) | 37 (45.1%) |
|  | In a relationship | 10 (16.9%) | 44 (31.4%) | 16 (20.0%) | 27 (32.9%) |
|  | Single | 15 (25.4%) | 28 (20.0%) | 13 (16.3%) | 17 (20.7%) |
|  | Does not want to answer/other | 0 (0.0%) | 2 (1.4%) | 0 (0.0%) | 1 (1.2%) |
| Share receiving other preventive treatments | | 44.1% | 44.3% | 28.7% | 19.3% |

*SD* standard deviation; *MHD* monthly headache days; *MMD* monthly migraine days.

^a^ The number are based on the full sample of respondents except for gender for the CGRP-mAbs group, other CM group, and LFEM group, which each have one missing reply, employment for the HFEM group, which has one missing reply, and civil status for the LFEM group, which has one missing reply.
